# Long-term effects of environmentally relevant concentrations of silver nanoparticles on major soil bacterial phyla of a loamy soil

**DOI:** 10.1186/s12302-018-0160-2

**Published:** 2018-08-31

**Authors:** Anna-Lena Grün, Christoph Emmerling

**Affiliations:** Department of Soil Science, Faculty of Regional and Environmental Science, University of Trier, Campus II, Behringstraße 21, 54296 Trier, Germany

**Keywords:** Silver Nanoparticles, Soil, *Bacteria phyla*, *Acidobacteria*, *Actinobacteria*, *Bacteroidetes*, *alpha*-*Proteobacteria*, *beta*-*Proteobacteria*

## Abstract

**Background:**

The growing production and use of engineered AgNP in industry and private households make increasing concentrations of AgNP in the environment unavoidable. Although we already know the harmful effects of AgNP on pivotal bacterial driven soil functions, information about the impact of silver nanoparticles (AgNP) on the soil bacterial community structure is rare. Hence, the aim of this study was to reveal the long-term effects of AgNP on major soil bacterial phyla in a loamy soil. The study was conducted as a laboratory incubation experiment over a period of 1 year using a loamy soil and AgNP concentrations ranging from 0.01 to 1 mg AgNP/kg soil. Effects were quantified using the taxon-specific 16S rRNA qPCR.

**Results:**

The short-term exposure of AgNP at environmentally relevant concentration of 0.01 mg AgNP/kg caused significant positive effects on *Acidobacteria* (44.0%), *Actinobacteria* (21.1%) and *Bacteroidetes* (14.6%), whereas *beta*-*Proteobacteria* population was minimized by 14.2% relative to the control (*p* ≤ 0.05). After 1 year of exposure to 0.01 mg AgNP/kg diminished *Acidobacteria* (*p* = 0.007), *Bacteroidetes* (*p* = 0.005) and *beta*-*Proteobacteria* (*p* = 0.000) by 14.5, 10.1 and 13.9%, respectively. *Actino*- and *alpha*-*Proteobacteria* were statistically unaffected by AgNP treatments after 1-year exposure. Furthermore, a statistically significant regression and correlation analysis between silver toxicity and exposure time confirmed loamy soils as a sink for silver nanoparticles and their concomitant silver ions.

**Conclusions:**

Even very low concentrations of AgNP may cause disadvantages for the autotrophic ammonia oxidation (nitrification), the organic carbon transformation and the chitin degradation in soils by exerting harmful effects on the liable bacterial phyla.

**Electronic supplementary material:**

The online version of this article (10.1186/s12302-018-0160-2) contains supplementary material, which is available to authorized users.

## Background

The increasing production and use of engineered silver nanoparticles (AgNP) in households, industry and agriculture [1–3] are leading to increased concentrations of AgNP in the environment. In Europe, Sun et al. [4] modelled a production of 32.4 tons year^−1^ nanosilver and predicted an annual increase of AgNP in the range of 1.2 ng/(kg year) to 2.3 ng/(kg year) for sediments and soils [4]. Soil is expected as the major sink for AgNPs released into the environment [5]. Considering the antimicrobial effects of AgNP and their concomitant Ag^+^ ions [1, 6, 7], an ecological risk assessment of AgNP is needed, but it requires understanding the long-term effects of environmentally relevant concentrations of AgNP on the soil microbiome.

Quite recently, we documented significant negative effects on soil microbial biomass and bacterial ammonia oxidizers after 1-year exposure to 0.01 mg AgNP/kg in a loamy soil [8]. The tested AgNP concentrations of 0.01–1.00 mg AgNP/kg were shown to significantly decrease the leucine aminopeptidase activity as well as the abundance of nitrogen fixing microorganism in our long-term investigation [8]. In addition, Hänsch and Emmerling [2] observed also a decrease in soil microbial biomass with increasing AgNP concentrations (0.0032–0.320 mg AgNP/kg) in a sandy loam after 4 months. These studies clearly demonstrate the significance of AgNP for soil functions like the organic matter transformation and the cycling of energy and nutrients [9, 10].

However, information about the impact of AgNP on the soil bacterial community structure is rare, although microbial communities are important and sensitive targets for determining the environmental hazards of AgNP [11]. Bacteria are the main performer of functional processes, which are integral for maintenance of healthy soil environments [12]. Some studies already documented the toxicity of AgNP to specific soil bacteria like *Pseudomonas putida* [13, 14] and *P. chlororaphis* [15] in single species studies. Moreover, analyses of the soil bacterial community composition after AgNP exposure were focused on phylum level. These revealed an AgNP intolerance by *Acido*- and *Actinobacteria*, whereas *Proteobacteria* and *Bacteroidetes* seemed to be unaffected or promoted by silver applications [16–19]. This might lead to devastating effects on acidobacterial driven organic carbon transformation [20] as well as on actinobacterial recycling of refractory biomaterials by decomposition and humus formation in soils [21]. As mentioned above, Shah et al. [22] observed not only a shift in the bacterial community structure after addition of 0.0625 mg AgNP/kg soil, but also a shift dependent on exposure time. The species richness sharply declined by prolonged incubation, which might be a response to the changing chemical state of silver in the soil [22].

In virtue of the numerous AgNP on the market, evaluating their potential ecotoxicological effects remains an essential challenge. The size, shape, surface-coating agent, charge and stability of AgNP are only some of the properties that can differ [1], and the characteristics of soils are also very distinct. Several studies indicated that the physicochemical characteristics of soils, like clay content, pH value and organic matter content correlated to various effects by AgNP and thus toxicity [23, 24]. Considering the significance of soil microbial communities for soil ecosystem function, such as plant growth carbon sequestration and degradation of xenobiotics [25] and the predicted increase of nanoparticle release into the environment, the aim of this study was to reveal the adverse long-term effects of AgNP on soil bacterial community structure. The study was conducted with an incubation period of 1 year using a loamy soil and AgNP concentrations in an environmentally relevant range (0.01–1.00 mg AgNP/kg soil). We measured the effects on a set of soil dominating bacterial phlya *Acidobacteria*, *Actinobacteria*, *Bacteroidetes*, *alpha*-*Proteobacteria* and *beta*-*Proteobacteria* by quantified marker genes using quantitative real-time PCR (qPCR).

## Methods

### Experimental setup

To assess the effects of silver nanoparticles (AgNP) on soil microbes, 20 kg of a loamy soil was sampled from the Ap horizon (0–30 cm depth) of an arable field cultivated with winter wheat in May 2013. The site is located at Helenenberg, NW of Trier, Germany (DD 49.8526°N, 6.5417°E). The soil can be characterized as a deeply developed haplic Stagno-Luvisol derived from Pleistocene eolian loess covering Middle Triassic limestone. The soil texture had a clay content of 17%–30%. The soil properties were previously described by Grün et al. [8]. After sampling, the soil was thoroughly sieved to < 2 mm and stored at 6 °C until further use.

Silver nanoparticles (AgNP), in the form of the certified reference material BAM-N001 (AgPure for analytical measurements [26] were purchased from Ras Materials (Regensburg, Germany). The AgPure stock solution (100 µg/ml) was homogenized by shaking by hand for 3 min according to manufacturer's instructions and then diluted stepwise using ultrapure water. Silver nitrate (AgNO_3_) was used as a positive control. Silver concentrations in the AgNO_3_ controls were the same as those in the AgNP treatments.

Before applying the test materials, the soil was moistened to a water content of 15.3%, which was equivalent to 40% WHC_max_ and incubated at 18 °C for 7 days. The application of the test materials was performed in petri dishes, each filled with soil equivalent to 25 g dry weight. Then, 1 mL AgPure or AgNO_3_ solutions, at different concentrations, was added in small drops onto the soil surface to obtain final concentrations of 10, 100 and 1000 µg/kg dry weight. Negative controls only received an application of ultrapure water. For each concentration, day and replicate, separate soil dishes were used. Subsequently, soils were extensively mixed by stirring with a spoon, and then they were transferred to plastic containers (Centrifuge Tubes, 50 mL, VWR, Darmstadt, Germany) and sealed by Parafilm^®^. They were incubated at (15 ± 4.5) °C in the dark for 1, 7, 14, 28, 180 and 365 days. The storage temperature slightly fluctuated according to the four seasons, with the highest temperatures at day 1 and day 365 (Summer) and the lowest temperatures at day 180 (Winter). Water evaporation was determined gravimetrically and then compensated with the addition of ultrapure water. Samples were finally stored at – 20 °C. For analyses, samples were defrosted by incubation overnight at 6 °C.

### DNA extraction

DNA extraction and purification were performed using the Genomic DNA from soil kit (Macherey–Nagel, Düren, Germany) according to the manufacturer’s instructions and stored at – 20 °C.

### Quantitative detection of bacterial phyla genes

The bacterial taxa *Acidobacteria* [27, 28], *Actinobacteria* [28, 29], *Bacteroidetes* [28, 30], *alpha*-*Proteobacteria* [31] and *beta*-*Proteobacteria* [32, 33] were quantified using the taxon-specific 16S rRNA qPCR assays listed in Table 1. All qPCR reactions were conducted on a thermal cycler equipped with an optical module (Analytik Jena, Jena, Germany). All samples were run in triplicate wells. Single qPCR reactions were prepared in a total volume of 20 µL. The InnuMix SYBR-Green qPCR Master-Mix was purchased from Analytik Jena (Jena, Germany). Primer concentrations were 10 pmol/µL, and amplification specificity was assessed by melting curve analysis and gel electrophoresis on an 1.5% agarose gel after qPCR. Standard curves were based on cloned PCR products from the respective genes [28] (Table 1).

**Table 1:** PCR primers and cycling conditions used for quantification of the different phyla

Phylum	Primer	Primer sequence 5′-3′	References	Organism for standard	Reaction mixture		Temperature programme		
*Acidobacteria*	Acid31	GAT CCT GGC TCA GAA TC	Barns et al. [27]	*Acidobacterium capsulatum*	Acid31	2 µL	95 °C	15 min	×1
					Eub518	2 µL	95 °C	15 s	×35
					Sybr Green	10 µL	55 °C	30 s	
	Eub518	ATT ACC GCG GCT GCT GG	Muyzer et al. [28]		DEPC H2O	2 µL	72 °C	30 s	
					DNA	4 µL	80 °C	30 s	
							60–95 °C		×1
*Actinobacteria*	Actino235	CGC GGC CTA TCA GCT TGT TG	Stach et al. [29]	*Arthrobacter crystallopoietes*	Actino235	1.5 µL	95 °C	15 min	×1
					Eub518	1.5 µL	95 °C	15 s	×35
					Sybr Green	10 µL	60 °C	30 s	
	Eub518	ATT ACC GCG GCT GCT GG	Muyzer et al. [28]		BSA 3%	0.4 µL	72 °C	30 s	
					DEPC H2O	2.6 µL	80 °C	30 s	
					DNA	4 µL	60–95 °C		×1
*α*-*Proteobacteria*	α682F	CNA GTG TAG AGG TGA AAT T	De Gregoris et al. [31]	*Bradyrhizobium japonicum*	α682F	2 µL	95 °C	15 min	×1
					908αR	2 µL	95 °C	15 s	×35
					Sybr Green	10 µL	61.5 °C	15 s	
	908αR	CCC CGT CAA TTC CTT TGA GTT			DEPC H2O	2 µL	72 °C	20 s	
					DNA	4 µL	60–95 °C		×1
*Bacteroidetes*	Cfb319	GTA CTG AGA CAC GGA CCA	Manz et al. [30]	*Flavobacterium aquatile*	Cfb319	2 µL	95 °C	15 min	×1
					Eub518	2 µL	95 °C	15 s	×35
					Sybr Green	10 µL	60 °C	30 s	
	Eub518	ATT ACC GCG GCT GCT GG	Muyzer et al. [28]		DEPC H2O	2 µL	72 °C	30 s	
					DNA	4 µL	80 °C	30 s	
							60–95 °C		×1
*β*-*Proteobacteria*	Eub338	ACT CCT ACG GGA GGC AGC AG	Overmann et al. [32]	*Alcaligenes faecalis*	Bet680	2 µL	95 °C	15 min	×1
					Eub338	2 µL	95 °C	15 s	×35
					Sybr Green	10 µL	55 °C	30 s	
	Bet680	TCA CTG CTA CAC GYG	Lane [33]		DEPC H2O	2 µL	72 °C	30 s	
					DNS	4 µL	80 °C	30 s	
							60–95 °C		×1

### Statistical analyses

All data were processed using IBM SPSS Statistics for Windows, Version 23.0 (IBM Corp., Armonk, USA). The obtained qPCR values (copy gene/kg dry soil) of negative controls (0.00 mg Ag/kg) were averaged for each phylum and day. Subsequent, the relative variation of a silver treated sample of one concentration and one sampling date were calculated as follow:

For the effect assessment of AgNP and Ag^+^ exposure in dependence of silver concentration, the applied test concentrations of AgNP and Ag^+^ were set as independent variables, whereas the relative variations of *Acidobacteria*, *Actinobacteria*, *alpha*-*Proteobacteria*, *Bacteroidetes* and *beta*-*Proteobacteria* were treated as dependent variables. For the effect assessment of AgNP and Ag^+^ exposure in dependence of sampling date, the sampling days (1 day–365 days) were set as independent variables, whereas the relative variations of different phyla at one test concentration were treated as dependent variables.

For each test concentration and day, 4 replicates per phylum were measured, and the average, median and standard deviation were calculated for these groups. In the following, groups were pre-evaluated for a normal distribution by the Shapiro–Wilk test. Variance homogeneity between the groups to be compared was calculated by the Levene test. The effects of different AgNP and Ag^+^ concentrations on 1 day and the effects of sampling date of one AgNP and Ag^+^ concentration, respectively, were compared with the ANOVA and Kruskal–Wallis test. Subsequent post hoc tests were performed by pairwise comparisons using the Dunn–Bonferroni or Games–Howell tests, depending on the requirements. Linear regression (least squares) and bivariate correlation (Pearson) were conducted for groups with homoscedasticity. For groups displaying heteroscedasticity, linear regression and bivariate correlation analyses were performed by weighted least squares (WLS).

## Results

### Test concentrations of AgNP and Ag^+^

For the effect assessment of AgNP and Ag^+^ exposure in dependence of silver concentrations, the applied test concentrations of AgNP and AgNO_3_ were compared to the relative variations of *Acidobacteria*, *Actinobacteria*, *alpha*-*Proteobacteria*, *Bacteroidetes* and *beta*-*Proteobacteria* at one sampling date.

### Short-term exposure: 1-day exposure

Short-term exposure of 1 day caused significant positive effects on *Acidobacteria* (43.95%), *Actinobacteria* (21.1%) and *Bacteroidetes* (14.6%) due to 0.01 mg AgNP/kg, whereas *beta*-*Proteobacteria* population was decreased by 14.15% relative to the control (Table 2). Results of Ag^+^ exposure in the form of AgNO_3_ revealed similar effects at 0.01 mg Ag/kg for *Acidobacteria* and *Bacteroidetes* (Table 2). At higher AgNP concentrations (0.1–1 mg AgNP/kg) *Acidobacteria*, *Actinobacteria* and *beta*-*Proteobacteria* were unaffected, whereas *Bacteroidetes* population increased by around 19% (*p* = 0.015). Higher Ag^+^ concentrations caused no effects on *Acidobacteria*, whereas the *Bacteroidetes* population was significantly stimulated by 40.0% (*p* = 0.000) relative to the control. The S16 rRNA copy numbers of *beta*-*Proteobacteria* significantly decreased at 0.1 and 1.0 mg Ag^+^/kg by 30.5% (*p* = 0.000) and 17.9% (*p* = 0.020), respectively (Table 2). The response of *Actinobacteria* was ambiguous (Table 2). *Alpha*-*Proteobacteria* showed no significant response to the tested AgNP and Ag^+^ concentrations on day 1.

**Table 2:** Summary of the relative variations of *Acidobacteria*, *Actinobacteria*, *alpha*-*Proteobacteria*, *Bacteroidetes* and *beta*-*Proteobacteria* compared to the untreated control related to AgNP and Ag^+^ exposure over 1 year in a loamy soil

Day	Conc. (µg/kg)	Copy gene number/g soil relative to the untreated control (%)																			
		*Acidobacteria*				*Actinobacteria*				*alpha*-*Proteobacteria*				*Bacteroidetes*				*beta*-*Proteobacteria*			
		AgNP		Ag^+^		AgNP		Ag^+^		AgNP		Ag^+^		AgNP		Ag^+^		AgNP		Ag^+^	
		*x*	*σ*	*x*	*σ*	*x*	*σ*	*x*	*σ*	*x*	*σ*	*x*	*σ*	*x*	*σ*	*x*	*σ*	*x*	*σ*	*x*	*σ*
1	0	0.00	7.67	0.00	7.67	0.00	4.87	0.00	4.87	0.00	9.60	0.00	9.60	0.00	5.58	0.00	5.58	0.00	5.92	0.00	5.92
	10	43.95*	13.40	39.85	22.08	21.13*	6.87	− 16.03	15.79	0.53	17.60	4.03	22.67	14.55	7.07	22.08*	7.34	− 14.15*	4.78	− 1.40	9.27
	100	− 1.38	25.03	3.43	34.96	2.15	8.22	− 26.37*	15.28	14.15	16.18	− 1.50	22.28	17.43*	10.60	39.95**	8.88	− 6.95	9.44	− 30.50**	6.98
	1000	5.68	17.95	31.40	18.47	0.38	15.94	36.55**	6.64	14.43	14.99	5.00	16.13	19.50*	6.53	37.30**	10.09	4.75	3.21	− 17.93*	4.73
7	0	0.00	12.28	0.00	12.28	0.00	7.02	0.00	7.02	0.00	7.22	0.00	7.22	0.00	5.42	0.00	5.42	0.00	7.49	0.00	7.49
	10	− 10.85	9.98	11.75	9.06	15.48	6.16	− 12.30	17.33	− 17.78*	3.84	− 6.38	12.64	24.25*	9.42	25.23**	6.29	− 28.35**	5.86	− 9.38	7.39
	100	− 17.45	16.12	3.53	5.95	− 0.30	7.80	/	/	− 0.43	3.05	10.38	10.39	17.63*	6.43	23.05**	4.09	− 11.80	9.08	− 1.43	7.55
	1000	− 11.63	12.28	0.98	10.74	15.80	13.04	− 10.68	13.16	− 5.43	6.68	− 7.05	15.42	24.95**	2.39	11.15*	3.82	− 24.15**	3.16	46.03**	7.64
14	0	0.00	9.28	0.00	9.28	0.00	3.48	0.00	3.48	0.00	9.21	0.00	9.21	0.00	4.69	0.00	4.69	0.00	10.69	0.00	10.69
	10	5.18	10.07	23.90**	8.50	12.88	12.71	10.70*	2.89	− 11.35	9.51	− 1.30	4.47	− 11.78*	1.21	− 1.43	2.71	− 10.80	1.71	− 2.10	1.54
	100	6.45	3.31	8.38	7.32	16.93	4.45	− 5.90	10.05	− 7.03	7.22	6.28	11.73	− 3.93	6.08	2.25	3.55	− 2.13	3.43	11.55	8.16
	1000	− 21.00*	12.45	12.23	5.11	− 8.65	11.53	− 27.62**	6.19	− 20.95*	10.01	5.05	10.67	− 16.43**	7.08	− 4.60	2.21	− 19.05	7.69	3.10	4.41
28	0	0.00	7.82	0.00	7.82	0.00	7.63	0.00	7.63	0.00	11.99	0.00	11.99	0.00	3.94	0.00	3.94	0.00	2.68	0.00	2.68
	10	− 1.28	2.25	10.28	5.55	− 20.00*	8.13	− 17.40	1.40	4.40	3.24	29.00**	6.45	4.78	2.00	− 4.40	4.67	− 14.28**	5.34	10.65	6.28
	100	− 4.93	3.11	4.23	10.24	− 21.60**	2.06	− 11.08	12.82	12.98	13.88	22.35*	3.98	0.25	1.27	− 0.90	7.32	− 12.48**	3.81	15.93*	7.93
	1000	2.45	2.93	6.55	10.96	− 19.53*	6.28	− 10.33	6.02	10.50	4.99	22.10*	4.15	3.05	1.35	9.83	4.18	11.30*	2.63	6.95	1.85
90	0	0.00	2.59	0.00	2.59	0.00	9.04	0.00	9.04	0.00	5.28	0.00	5.28	0.00	3.26	0.00	3.26	0.00	4.64	0.00	4.64
	10	− 31.33**	3.49	− 17.88*	8.64	− 22.40*	12.68	− 25.30*	4.74	− 20.38**	5.39	− 18.08**	3.77	− 21.98**	3.05	− 19.05*	2.23	− 30.10**	1.53	− 22.68**	1.83
	100	6.50	5.04	− 30.35**	4.66	− 23.88*	11.84	− 28.00**	9.43	− 20.15**	3.62	− 20.40**	2.99	− 22.55**	4.11	− 17.83*	7.44	− 33.23**	5.30	− 13.05*	4.00
	1000	− 2.85	6.32	− 31.53**	12.22	− 35.20**	4.20	− 15.15	11.50	− 24.53**	6.42	− 12.08*	5.06	− 25.15**	4.51	− 21.05*	11.34	− 20.03**	4.11	− 28.70**	4.61
180	0	0.00	9.76	0.00	9.76	0.00	12.16	0.00	12.16	0.00	8.09	0.00	8.09	0.00	8.29	0.00	8.29	0.00	5.63	0.00	5.63
	10	15.83	12.47	− 6.53	10.25	− 18.05	5.96	− 26.00*	6.91	− 14.70	8.82	− 26.70*	11.35	− 10.23	3.73	− 22.43**	6.32	− 19.68**	2.90	− 14.35**	4.77
	100	4.23	14.08	− 14.55	8.45	− 34.68	22.77	− 29.08**	9.60	− 9.85	8.52	− 17.48	11.11	− 14.40	3.61	− 30.30**	5.18	− 29.23**	6.35	− 14.55**	3.75
	1000	3.75	8.24	− 28.25**	4.46	− 2.50	19.79	− 20.33*	2.70	− 19.50*	4.64	− 40.65**	6.05	− 10.60	1.69	18.73*	7.04	− 19.30**	3.23	− 3.05	3.77
365	0	0.00	4.95	0.00	4.95	0.00	8.06	0.00	8.06	0.00	1.68	0.00	1.68	0.00	2.07	0.00	2.07	0.00	1.95	0.00	1.95
	10	− 14.45**	2.77	− 5.78	4.02	4.83	9.46	− 2.65	2.37	− 1.35	3.38	15.60**	4.59	− 10.10**	3.74	− 25.78*	6.30	− 13.85**	1.51	− 14.80**	3.52
	100	− 7.48	5.86	− 3.78	7.11	6.63	12.52	− 11.80	5.66	− 6.18	3.37	1.03	2.39	− 20.70**	2.43	− 2.18	4.22	− 17.58**	1.78	− 28.08**	2.51
	1000	− 7.10	5.13	− 2.95	6.10	− 7.93	8.00	− 24.13**	7.19	− 0.03	3.47	− 4.03	3.10	− 23.15**	4.07	− 16.45*	5.10	− 27.10**	2.23	− 16.85**	4.43

Underlined values highlight significant relationships between groups of different concentrations of one agent (AgPure or Ag^+^) (ANOVA, *p* ≤ 0.05). * Indicates significant results of pairwise comparisons (*p*) of the post hoc test related to negative control (*p* ≤ 0.05). ** Indicate significant results of pairwise comparisons (*p*) of the post hoc test related to negative control (*p* ≤ 0.005). *x* = mean, *σ* = standard deviation

### Mid-term exposure: 14–28 days of exposure

After 1 week, AgNP and Ag^+^ exposure caused similar significantly positive effects on *Bacteroidetes* like on day 1 (*p* = 0.000) (Table 2). *Alpha*- and *beta*-*Proteobacteria* were significantly diminished by 17.8% (*p* = 0.011) and 28.4% (*p* = 0.000) at 0.01 mg AgNP/kg. Furthermore, *beta*-*Proteobacteria* population decreased due to 1.0 mg AgNP/kg exposure on day 7 (*p* = 0.002), whereas 1.0 mg Ag^+^/kg increased the *beta*-*proteobacterial* population by 46.0% (*p* = 0.000) relative to the control. *Acido*- and *Actinobacteria* were unaffected at this sampling point in regard to silver treatments.

In the following weeks, both AgNP and Ag^+^ caused notable effects on *Acidobacteria* and *Actinobacteria* (Table 2). At day 14, AgNP reduced the populations of *alpha*-, *beta*-*Proteobacteria* and *Bacteroidetes* (*p* < 0.05), whereas these groups were unaffected due to Ag^+^. After 1 month, exposure of AgNP caused no further effects on *alpha*-*Proteobacteria* and *Bacteroidetes*. *Beta*-*Proteobacteria* population was significantly minimized at 0.01 (*p* = 0.001) as well as 0.1 mg AgNP/kg (*p* = 0.003) and, in contrast, enlarged by 1.0 mg Ag^+^/kg (*p* = 0.007) (Table 2).

### Long-term exposure: 90–365 days of exposure

At day 90, AgNP and Ag^+^ exposure led to significant decreases of all phyla populations (Table 2). The relative variations to the negative control documented tendentially stronger or equal impacts of AgNP in relation to Ag^+^ (Table 2). For instance, tested nanoparticulate silver concentrations on day 90 caused an averaged decrease by 27.2% of actinobacterial population, whereas ionic silver concentrations caused an averaged decrease by 22.8%. With exception of the *beta*-*Proteobacteria* population, this trend inversed at day 180 and Ag^+^ influenced population sizes more intensive compared to AgNP (Table 2). Now, AgNP had no more effects on *Acidobacteria*, whereas Ag^+^ reduced *Acidobacteria* population by averaged 16.4%.

At the end of the long-term experiment at day 365, effects due to AgNP were still remarkable. As little as 0.01 mg AgNP/kg diminished *Acidobacteria* (*p* = 0.007), *Bacteroidetes* (*p* = 0.005) and *beta*-*Proteobacteria* (*p* = 0.000) by 14.5, 10.1 and 13.9%, respectively (Table 2). The equal concentration of Ag^+^ caused no effect, decrease by 25.78% and nearly the same decrease of 14.8% for the groups, respectively (Table 2). Finally, the averaged effects of the tested concentrations were the highest for *beta*-*Proteobacteria* and *Bacteroidetes*, which showed significant decline of 19.5% by AgNP and 19.9% by Ag^+^ as well as 18.0% by AgNP and 14.8% by Ag^+^, respectively. *Actino*- and *alpha*-*Proteobacteria* were statistically unaffected by AgNP treatments after 1-year exposure (Table 2), whereas Ag^+^ treatments significantly reduced or stimulated their populations, respectively (Table 2).

### Exposure time of AgNP and Ag^+^

**Fig. 1:**
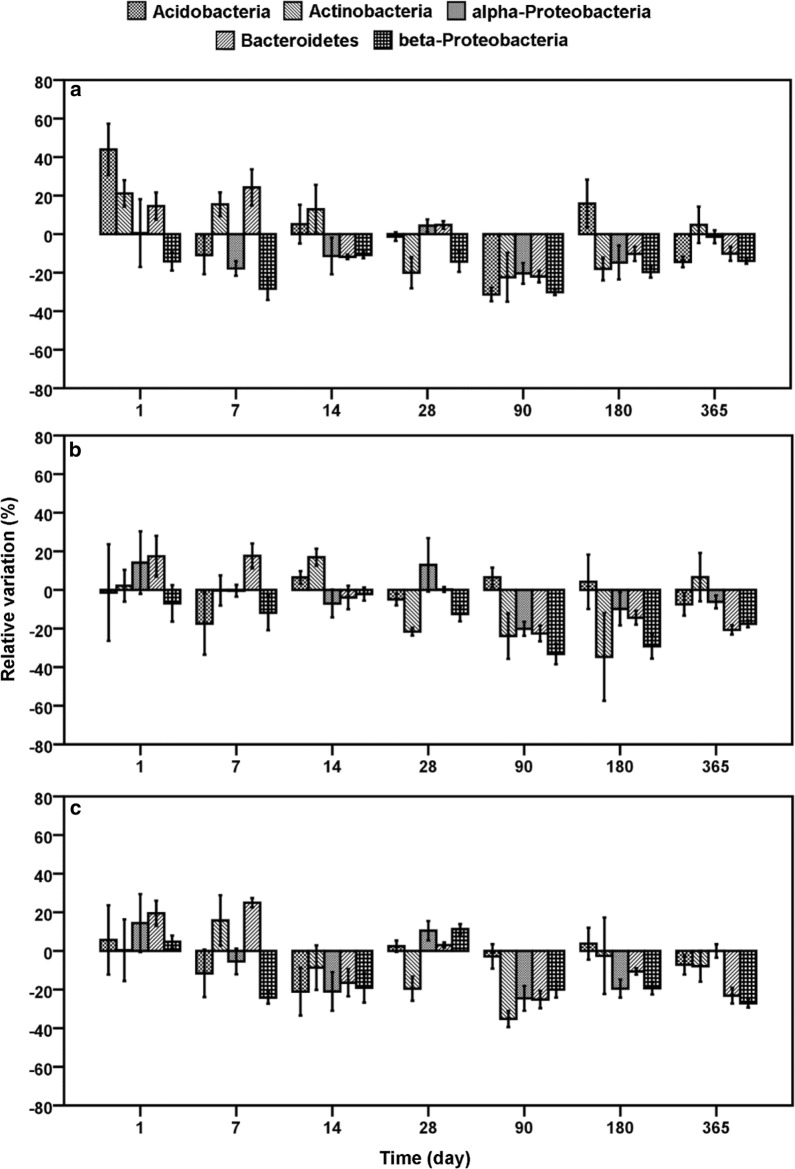
Relative variations of *Acidobacteria*, *Actinobacteria*, *alpha*-*Proteobacteria*, *Bacteroidetes* and *beta*-*Proteobacteria* compared to untreated control related to time at different AgNP concentrations. **a** 0.01 mg AgNP/kg, **b** 0.10 mg AgNP/kg, **c** 1.00 mg AgNP/kg

**Fig. 2:**
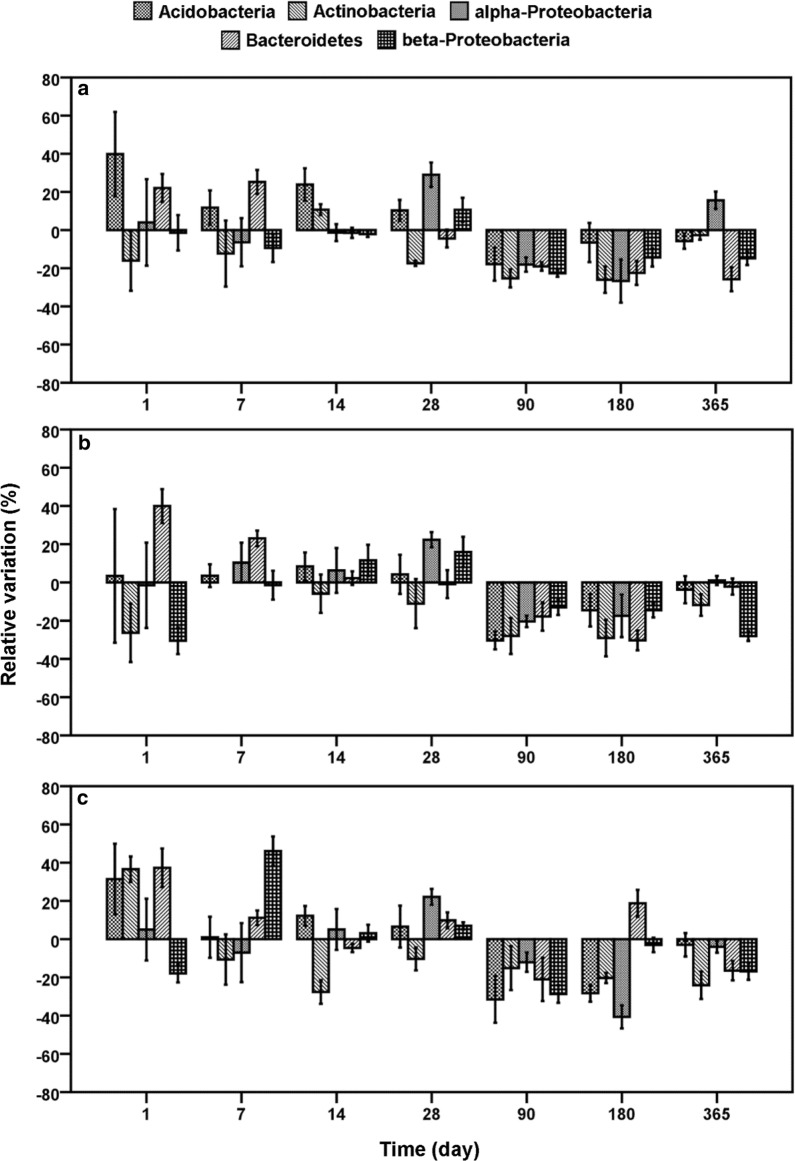
Relative variations of *Acidobacteria*, *Actinobacteria*, *alpha*-*Proteobacteria*, *Bacteroidetes* and *beta*-*Proteobacteria* compared to untreated control related to time at different Ag^+^ concentrations. **a** 0.01 mg Ag^+^ kg^−1^, **b** 0.10 mg Ag^+^ kg^−1^, **c** 1.00 mg Ag^+^ kg^−1^

For the effect assessment of AgNP and Ag^+^ exposure in dependence of sampling date, the sampling days (1 day–365 days) were compared to the relative variations of the different phyla at one test concentration. In Figs. 1 and 2, the temporal progress of relative variations for each phylum at different concentrations, 0.01 (a), 0.10 (b) and 1.00 (c), of AgNP and Ag^+^ was visualized.

Hypothesis tests revealed that the exposure time of AgNP and Ag^+^ had consistent significant effects on the relative variations of the bacterial population sizes of each phylum at each concentration (*p* < 0.025). For example, AgPure treatment of 0.01 mg AgNP/kg soil created different effect characteristics on the relative variations of *Actinobacteria* in dependence on sampling date. Here, ANOVA analysis indicated a *p* value of 0.000 between the groups of 1, 7, 14, 28, 90, 180 and 365 days. Results of the post hoc test are shown in Table 3.

**Table 3:** Pairwise comparisons (*p*) of the post hoc test for comparing the relative variations of *Actinobacteria* relative to untreated control at 0.01 mg AgNP/kg in dependence of sampling day

Sampling date [d]	1	7	14	28	90	180	365
1		/	/	0.000	0.000	0.000	/
7	/		/	0.000	0.000	0.001	/
14	/	/		0.001	0.000	0.002	/
28	0.000	0.000	0.001		/	/	0.022
90	0.000	0.000	0.000	/		/	0.009
180	0.000	0.001	0.002	/	/		0.045
365	/	/	/	0.022	0.009	0.045	

Only significant pairs were stated by their p value. In case of “/” no significant comparison could be observed

Linear regression and correlation analysis showed only one moderate correlation (*r*^*2*^ = 0.537) between the relative variation of the rRNA copy number in relation to the untreated control and the sampling date for *Bacteroidetes* exposure to 0.01 mg Ag^+^/kg (*p* = 0.000). The linear regression and correlation analyses to reveal potential relationships between the relative variation and sampling time for the remaining phyla showed only very weak significant correlations or were not significant (data not shown). Thus, the majority of the temporal progresses of relative variations for each phylum at the different concentrations did not follow linear relationships about the exposure period of 1 year.

However, linear regression and correlation analysis between the relative variation of the rRNA copy number and the sampling dates between day 90 and 365 revealed 18 out of 30 possible significant linear regressions (*p* < 0.05) (Table 4). Especially in case of AgNP, there seemed to be a strong time dependence of toxicity. Only in case of *Acidobacteria*—0.1 mg AgPure/kg and *Bacteroidetes*—0.01 mg Ag^+^/kg, the relationships were negative correlated. Regarding to *Acidobacteria, r*^2^- and *p*-values of 0.356 and 0.040 were indicative of a weak correlation. In the cases of the majority of the linear regressions, the toxicity of silver agents decreased significantly with time.

**Table 4:** Results of Pearson correlation analysis for comparing the relative variations of the phyla at different AgNP and Ag^+^ concentrations in dependence of exposure time between 90 and 365 days

	µg/kg	*Acidobacteria*		*Actinobacteria*		*Alpha*-*Proteobacteria*		*Bacteroidetes*		*Beta*-*Proteobacteria*	
		*r* ^*2*^	*p*	*r* ^*2*^	*p*	*r* ^*2*^	*p*	*r* ^*2*^	*p*	*r* ^*2*^	*p*
AgPure	10	/	/	0.646	0.002	0.682	0.001	0.441	0.018	0.818	0.000
	100	0.356*	0.040*	0.419	0.023	0.472	0.014	/	/	0.707	0.001
	1000	/	/	/	/	0.842	0.000	/	/	0.508	0.009
Ag^+^	10	/	/	0.758	0.000	0.670	0.001	/	/	/	/
	100	0.699	0.001	0.465	0.015	0.694	0.001	0.421*	0.023*	0.788	0.000
	1000	0.723	0.000	/	/	/	/	/	/	/	/

Negative correlations were marked by a star. In case of “/” no significant comparison could be observed

## Discussion

Soil bacteria are involved in major soil processes, such as humification, recycling, mineralization of organic matter and stabilization of soil structure [25]. Although linking members of bacterial communities in soils with their function has proven to be still difficult through their phylogenetically diversity [34], not cultivability [35] and functional redundancy [36], some substantial soil functions could be dedicated to specific soil bacteria phyla. For instance, *beta*-*Proteobacteria* were linked to autotrophic ammonia oxidation (nitrification) [37], *Actinobacteria* to decomposition and humus formation [21], *Acidobacteria* to organic carbon transformation [20, 38, 39], *alpha*-*Proteobacteria* to CO_2_-fixation, carbon degradation and sulphur cycling [38], and *Bacteroidetes* to chitin degradation [38] in soils.

Furthermore, certain bacteria phyla could be used as indicators of nutrient status, soil acidity, soil pollution and changes of other environmental factors [40, 41]. Here, we discuss the abundance of five different bacterial phyla in dependence of different nanoparticulate and ionic silver concentrations, as well as of the exposure time.

### Short-term exposure: 1-day exposure

Short-term exposure of Ag^+^ caused predominantly stronger effects on the investigated populations compared to AgNP (Table 2). These distinct effect responses in case of the two silver forms were indicative of their time-dependent reactivity in the complex physicochemical soil system. Assuming that Ag^+^ ions released by AgNP caused the effects on day 1, the dissolution of AgNP after short-term exposure took more time in contrast to Ag^+^ released by AgNO_3_. Several studies [8, 42–44] documented a slow and progressive increase in AgNP toxicity with time and assumed a time-dependent enlargement of silver ions due to slow dissolution. Dissolution of AgNP in soils is influenced by oxidation [45] and surface blocking induced by organic matter and mineral phase constituents [42]. Here, dissolution of the AgNP was certainly, because the high concentration of divalent cations, such as Ca^2+^ und Mg^2+^, in the Stagno-Luvisol promoted AgNP dissolution, resulting in the displacement of Ag^+^ ions from the nanoparticle surface [46]. Moreover, the dissolution hypothesis was also supported by the low concentrations of AgNP in the test soil, their polyacrylate stabilization and the high pH value of soil that could have prevented initial aggregation and agglomeration of AgNP [47]. Apart from that, soil inhibits the release of Ag^+^ ions due to organic matter coatings [48]. Consequently, the combination of dissolution and stability of AgNP led to lower effect strength.

Both AgNP and Ag^+^ chiefly provoked similar effects on *Acidobacteria*, *Bacteroidetes* and *beta*-*Proteobacteria in the respective silver concentration*. *Acidobacteria* and *Bacteroidetes* were generally stimulated, whereas *beta*-*Proteobacteria* was primarily diminished by silver addition. The effect strengths were in general higher for Ag^+^ compared to AgNP. Actually, *Acidobacteria* are known for their AgNP intolerance in soils [16, 17, 19, 49]. Here, they were significantly stimulated after 1-day exposure to environmentally relevant concentration of 0.01 mg AgNP/kg. With increasing concentrations, the effect strength diminished (Table 2). The extrusion of heavy metal ions by efflux systems, the reduction into less toxic oxidative states and the production of extracellular proteins or polysaccharides are common bacterial metal resistance mechanisms [50–52]. Ward et al. [39] reported a variety of ion channels, excretion of extracellular slime and resistance-nodulation-cell division transporter system for *Acidobacteria*, which could explain the acidobacterial AgNP tolerance. Furthermore, Yang et al. [53] found silver tolerance genes in acidobacterial genera. In addition, hormone-like responses to low silver concentrations were reported as a reason for stimulatory effects due to AgNP exposure [54, 55]. The previously observed contrasting acidobacterial AgNP intolerance by e.g. Juan et al. [16] or McGee et al. [17] might be the result of their high test concentrations of 10 to 100 mg AgNP/kg soil, which could prevent metal resistance mechanisms and hormone-like responses. The stimulation of *Bacteroidetes* due to nanoparticulate and ionic silver addition was in agreement with previous observations [16, 17, 19, 53, 56]. They exhibit also silver resistance genes [53]. Members of *beta*-*Proteobacteria* harbour likewise silver resistance genes [53], which could be the underlying reason for non-observable effects at concentrations higher than 0.01 mg AgNP/kg. Apart from that, 0.1 and 1.0 mg Ag^+^/kg significantly lowered the beta-proteobacterial population after short-term exposure (Table 2). Thus, it seemed to be more probable that the effects were caused by Ag^+^ again, and dissolution of AgNP needed more time compared to AgNO_3_. At the environmentally relevant concentration of 0.01 mg AgNP/kg, a significantly decrease of *beta*-*Proteobacteria* by 14.2% (*p* = 0.047) was observed, whereas Ag^+^ caused no effects. Here, it might be possible that AgNP directly impacted *beta*-*Proteobacteria* by effecting cell walls and membranes, the production of reactive oxygen species (ROS) and modifications of nucleic acids. Furthermore, the decrease of *beta*-*Proteobacteria* at 0.01 mg AgNP/kg could be an evidence for an expression threshold of silver resistance genes. Because AgNP showed slower dissolution, the Ag^+^ concentration was not sufficient for the expression of silver resistance genes and Ag^+^ could inhibit the respiratory chain, collapse the proton motive force, and influence the phosphate uptake and DNA molecules [57] of *beta*-*Proteobacteria*.

*Alpha*-*Proteobacteri*a were statistically unaffected due to AgNP and Ag^+^ exposure after 1 day. Here, also silver resistance genes could be the underlying reason as well as common bacterial resistance mechanisms [50–52].

The stimulation of *Actinobacteria* at low AgNP concentrations and the diminution at the same Ag^+^ concentrations (Table 2) were again an evidence for slow AgNP dissolution and Ag^+^ release. The promotion of *Actinobacteria* at 1.0 mg AgNP/kg was indicative for actinobacterial silver resistance mechanism, which needed a threshold concentration to become active. Vasileiadis et al. [58] documented silver resistance possibility for some actinobacterial members.

### Mid-term exposure: 14–28 days of exposure

In contrast to the hypothesis of slow and progressive enlargement of AgNP toxicity with time, the effect strength of AgNP decreased at mid-term exposure between day 7 and 28 (Table 2). The same was observed for Ag^+^ in the form of AgNO_3_ (Table 2). However, the effect strength of both silver forms became more similar and Ag^+^ showed only small higher effects on the bacterial phyla compared to AgNP. This supports the dissolution hypothesis of Ag^+^ by AgNP, which took more time. The smaller effect strength might be due to interactions of the silver species with the soil compartment. In the environment, AgNP is relatively susceptible to transformations (e.g. changes in aggregation and oxidation state, dissolution, sulfidation, sorption of inorganic and organic species), thus modifying their physical and chemical properties and behaviour [59, 60]. Moreover several studies indicated that the physicochemical characteristics of soils correlated to various effects by AgNP and thus toxicity [23, 24]. It is likely that the short-term effects were observed as a result of the initial release of bioavailable Ag^+^ and the AgNP, which could be reduced at later time points due to interactions of the silver species with organic matter, clay minerals or pedogenic oxides. According to previous investigations, high clay content (approximately 30%) of the Stagnic-Luvisol and high content of organic carbon (2.9%) could lead to a higher retention of AgNP and Ag^+^ ions a few days after the initial contamination [48, 61–63]. Furthermore, self-protection mechanisms, like the production of extracellular proteins or polysaccharides of the soil microbiome, could neutralize toxic ions or cap AgNP [51, 52]. Also, resilience mechanisms, such as fast growth rates, metabolic flexibility, physiological tolerance [36] and/or cryptic growth [64], might also be possible explanations for the limited effects on the bacterial phyla in the soil. These interactions between soil, silver agents and microbial community influenced the toxicity to such an extent that a linear relationship between time and toxicity could not be observed (Figs. 1 and 2). Nevertheless, the pairwise comparisons of post hoc tests for comparing the relative variations relative to untreated control at different silver concentrations in dependence of sampling day distinctly proved a strong relationship between exposure time and toxicity.

Focusing on the individual bacterial groups revealed overall no significant effects on *Acidobacteria* after exposure to nanoparticulate as well as ionic silver for 7 to 28 days (Table 2). Apart from the already stated acidobacterial silver resistance mechanisms, a shift in their community structure towards more silver-tolerant species could be probable [53]. Additionally, Ulrich and Becker [65] documented that *Acidobacteria* seemed to participate frequently in the shifting of community structures that result from soil property changes. Only 1.0 mg AgNP/kg caused a significantly decrease of Acidobacteria after 14 days (*p* = 0.049), but the acidobacterial population recover until day 28 probable due to resilience mechanisms.

*Actinobacteria* seemed also statistically unaffected by Ag^+^ and AgNP after 1-week exposure, but commencing with day 14, significant decreases of actinobacterial communities were observed. The effect strengths for both silver species were very similar, leading to the assumption of Ag^+^ as toxicological agent. In contrast to the stated actinobacterial silver resistance possibility, Vasileiadis et al. [58] as well as Juan et al. [16] noticed sensitivity against silver, which could be confirmed in our study. The copy number of *alpha*- and *beta*-*Proteobacteria* as well as of *Bacteroidetes* relative to the untreated controls showed distinct and changeable responses to mid-term silver additions (Table 2). These periodic and concentration-dependent fluctuations could be attributed to the physiological and ecological diversity of the members of one phylogenetic group as well as to the soil and agent interactions in a complex space–time framework. Nevertheless, after 1-month exposure, *Bacteroidetes* were unaffected by AgNP and Ag^+^ (Table 2). McGee et al. [17] monitored a similar resistance of *Bacteroidetes* after exposure to 50 mg AgNP (20 nm)/kg soil after 30 days in soil. The *alpha*-*Proteobacteria* population was also unaffected by AgNP, but showed a significantly growth due to Ag^+^ treatment (Table 2). Previous studies documented alpha-proteobacterial increase due to low metal concentrations [66, 67]. Furthermore, *alpha*-*Proteobacteria* are known as ecological most diverse group [40]. Therefore, a shift within the alpha-proteobacterial population due to silver exposure could have unpredictable effects on CO_2_ fixation, carbon degradation and sulphur transformation by their colonization of new ecological niches in consequence of silver emission into soils.

The rise of beta-Proteobacteria due to Ag^+^ (Table 2) was consistent with our previous study [8], indicating a stimulation of beta-proteobacterial ammonia oxidizers by hormone-like responses to low silver concentrations [54, 55] The stimulation of beta-proteobacterial ammonia oxidizers on the one hand and the sensitivity of the overall *beta*-*Proteobacteria* population due to AgNP on the other hand (Table 2) were indicative for distinct susceptibilities among the members of this class. While the amoA-harbouring genera *Nitrosomonas* and *Nitrosospira* seemed to be more AgNP tolerant, other genera or species of this group were very sensitive. A more precise study is essential for resolving this observation.

### Long-term exposure: 90–365 days of exposure

Similar to our observations concerning the impact of AgNP and Ag^+^ on microbial biomass, enzyme activity and functional genes involved in the nitrogen cycle of loamy soil [8], long-term exposure starting at exposure day 90 led to significant ecotoxicity (Table 2). Ageing of both silver forms and their slow return to the biological soil system presented a continuous sinking of bioavailable silver. As already mentioned, an increase in AgNP toxicity with time can be linked to time-dependent enlargement of silver in soil pore water due to dissolution [42, 68]. The interplay of nanoparticulate and ionic silver effect strength demonstrated the toxicity of Ag^+^ ions, if silver nitrate caused stronger effects, and if soil conditions inhibit Ag^+^ release, soil-aged AgNP may still act as a sink for bioavailable silver, as Ag^+^ ions absorbed on the particle surface may be released into the soil system [48]. Interestingly, the toxicity of AgNP as well as Ag^+^ predominantly decreased significantly with ongoing exposure time between 90 and 365 days (Table 2, Table 4). There seemed to be a shock load of silver on day 90 to which the bacterial groups were not immediately prepared. Based on our data, we could only speculate about this event. The intricacy of soil and agent interactions in the complex space–time framework influenced the silver toxicity mechanisms. Small-scale bioavailability, chemical alterations and possible transformations (aggregation, dissolution, sulfidation, sorption) of AgNP and Ag^+^ [59, 69] in the Luvisol are only a few possible physicochemical causes. Nevertheless, the decline of silver toxicity on the bacterial phyla at day 180 and 365 indicated again silver resistance and resilience mechanisms as described above. It might be assumed that after short- and mid-term adaption to the silver contamination as well as the positioning of the silver species in the soil system, the bacterial population might have lost its silver tolerance and were unanticipatedly shocked the return of silver toxicant at day 90 resulting in strong population reductions. Not until then, bacteria could replenish their arsenal of defense mechanisms and the toxicity of silver particles could be reduced. Nevertheless, the silver effects were stronger compared to short- and mid-term exposure, indicating indeed soil as a continuous sink of bioavailable silver.

## Conclusion

Changes of the relative abundance of *Acidobacteria*, *Actinobacteria*, *alpha*- and *beta*-*Proteobacteria* as well as for *Bacteroidetes* along AgNP concentrations ranging from 0.01 to 1 mg AgNP/kg soil over a long-term period of 1 year using a loamy soil were analysed by 16S rRNA qPCR technique. After 1-year exposure, we found that the abundances of *Acidobacteria, Bacteroidetes* and *beta*-*Proteobacteria* were significantly diminished after long-term exposure to environmentally relevant concentration of 0.01 mg AgNP/kg loamy soil. *Actino*- and *alpha*-*Proteobacteria* were statistically unaffected by AgNP treatments after 1-year exposure. Thus, even very low concentrations of AgNP may cause disadvantages for the autotrophic ammonia oxidation (nitrification), the organic carbon transformation and the chitin degradation in soils by exerting harmful effects on the liable bacterial phyla.

Furthermore, the statistically significant relationships between silver toxicity and exposure time presented loamy soils as a sink for silver nanoparticles and their concomitant silver ions.

## Additional file

**Additional file 1.**
